# Spasmodic Torticollis

**Published:** 1895-07-20

**Authors:** A. Hanbury Frere


### Medical Progress and Hospital Clinics.

[*The Editor will be glad to receive offers of co-operation and contributions from members of the profession. All letters should be addressed to* The Editor, The Lodge, Porchester Square, London, W.]

### SPASMODIC TORTICOLLIS.

By A. Hanbury Frere, M.B.

Functional disorders, the nature and causes of which are still obscure, are often exceedingly interesting. Considered as links in the chain of nervous derangements they may be very instructive. The condition known as spasmodic torticollis, or wryneck, is one of these. Having regard to the symptoms and course of this affection, we place it, provisionally, among the spasmodic neuroses, until such time as our knowledge shall suggest to us a better classification.

Allied, on the one hand, to histrionic spasm, which is more or less of the nature of a "bad habit," and, on the other, to "fatigue" or "handicraft spasm," spasmodic torticollis has been ascribed to many different causes. The treatment has, until somewhat recently, been very unsatisfactory. Whilst internal medicaments may prove utterly powerless to influence its course, a cure may follow the most diverse remedies, and each in turn fail "more often than it succeeds." Indeed, neither drugs, nor external applications, nor electricity appear to be of any permanent value. It is true that massage has proved beneficial, but only in comparatively recent cases. Attention was thus turned to the affected muscles, and analogy suggested the propriety of dividing the contracted fibres either subcutaneously or by the open method. This necessitated fixing the head in its proper position by means of some apparatus, but such a procedure is extremely irksome to the patient and the results have not been entirely satisfactory. Neurectomy and nerve stretching afford but temporary relief. There remains, therefore, but one method of treatment that promises any hope of lasting benefit, and that is neurectomy, or "removing a considerable portion of the nerve or nerves supplying the affected muscles." In most cases it is the spinal accessory nerve that is affected, causing spasm of the sterno-mastoid muscle, with the result that "the head is bent forward, the chin turned to the opposite side, and elevated." If the trapezins muscle is also affected the position is the same, but "the head is drawn more backward and sideways." In some cases it is said that the tendency to rotation of the head may be overcome by contraction of the posterior rotator muscles of the same side, but as a rule it appears that the posterior rotator muscles are affected on the opposite side, thus increasing, if anything, the rotation caused by the contraction of the sterno-mastoid. The cure of this disease may, therefore, necessitate something more than the removal of a portion of the spinal accessory nerve, for in addition we may require to resect the sub-occipital, and succeeding posterior primary divisions of the cervical nerves, the mention of which takes us back to the good old days in the dissecting room, engaged in that often too hurried "dissection of the back," or absorbed in the mysteries of the "posterior triangle of the neck." In torticollis changes may take place in the orbital muscles so that the eyes may accommodate themselves to the altered position of the head, and maintain the horizontal line of the retinæ at right angles to the middle line of the body. This, according to Hübscher, may increase the difficulty of cure, so great is the tendency to return to the malposition, in order to assist the weakened eye muscles to keep the eyeballs horizontal. There are many interesting features connected with this disease, and we cannot, perhaps, do better than give the conclusions arrived at by Richardson and Walton from a study of this distressing complaint. (American *Journ. Med. Sci.*, Jan., 1895.) They find that palliative treatment, whether by drugs, apparatus, or electricity will rarely prove successful in well-established cases. In comparatively recent cases massage may prove of value, but resection affords practically the only rational remedy. Operation on the spinal accessory nerve may afford relief, even if other muscles than the sterno-cleido-mastoid are affected; on the other hand, the affection previously limited to the sterno-mastoid may spread to other muscles in spite of this operation. The head can be held erect even after the most extensive resection. The most common combination of spasm is that involving the sterno-mastoid on the one side and the posterior rotators on the other, the head being held in the position of sterno-mastoid spasm with the addition of retraction through the greater power of the posterior rotators. It seems advisable in most cases to give preference to the resection of the spinal accessory as the preliminary procedure.

